# Traumatic Urethral Injury without Pelvic Fracture in an Adult Female

**DOI:** 10.1100/tsw.2010.29

**Published:** 2010-02-17

**Authors:** Nathan A. Bockholt, Kenneth G. Nepple, Charles R. Powell, Karl J. Kreder

**Affiliations:** Department of Urology, University of Iowa, Iowa City, IA, USA

**Keywords:** urethra, wounds and injuries, trauma, pelvis, female

## Abstract

A 23-year-old female was involved in a motor vehicle collision with multiple injuries, including a right acetabular fracture, but no pelvic fracture. Urology consultation was obtained due to difficulty placing a urethral catheter. Examination revealed a longitudinal urethral tear with vaginal laceration extending 2 cm from the urethral meatus proximally toward the bladder neck. The longitudinal urethral tear was repaired primarily. Traumatic female urethral injury in the absence of a pelvic fracture is an exceedingly rare occurrence.

## CASE PRESENTATION AND MANAGEMENT

A 23-year-old female was a restrained driver in a head-on, motor vehicle collision at highway speed. On initial presentation, a Foley catheter was placed with return of gross blood and no urine. Radiographic imaging showed multiple injuries, including bilateral femur fractures and a right acetabular fracture. However, no pelvic fracture was present. On CT, a left superior pole kidney infarct was present and the Foley catheter balloon appeared to be in the vagina (Fig. 1). Urology consultation was obtained and a genitourinary exam showed a Foley catheter entering the urethral meatus; however, on manual examination, the catheter and balloon were palpated 2 cm inside the vagina. A large, full-thickness, urethral tear was present and the inside of the bladder could be palpated anterior to the catheter. The catheter was manipulated back into the bladder, producing return of urine.

The urethral injury required operative repair. The patient required concomitant orthopedic surgery with bilateral femur external fixation, which was performed first to allow the patient to be positioned in dorsal lithotomy. In the operating room on exam, the distal 2 cm of the urethra was intact. A traumatic, longitudinal, full-thickness, urethral vertical tear with vaginal laceration extended for 5 cm and involved the bladder neck. Location of the tear was midline without evidence of ureteral injury. Primary urethral repair was performed vaginally with closure of the urethra and bladder mucosa with interrupted 3-0 chromic sutures. The muscularis over the bladder and urethra was closed in a second layer of running locking 2-0 Vicryl. The vaginal mucosa was then closed as a third layer over the muscularis with a running locking 2-0 Vicryl, with an attempt being made to offset the suture line from the muscular closure. A Foley catheter was left to straight drainage. The urethral catheter was removed 6 weeks postoperatively and repair was intact. The patient was seen 2 months after the repair and was continent of

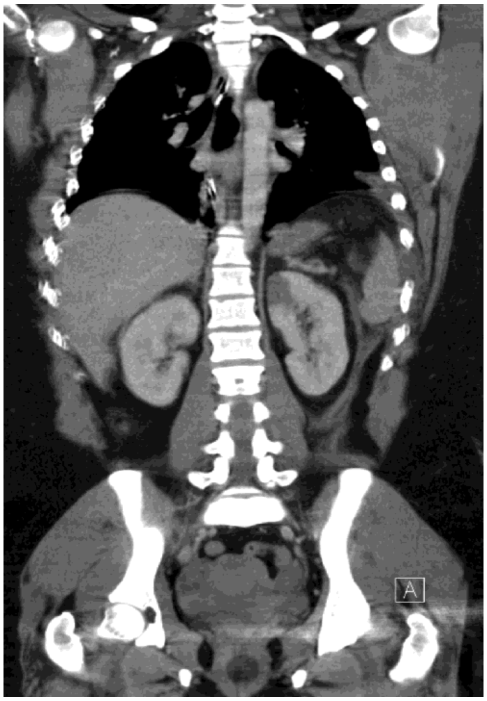

**FIGURE 1.** CT demonstrating inferior displacement of the Foley catheter balloon in the vagina.

urine. She did not report urgency or frequency. There was no breakdown in the wound and no vaginal discharge. She did have one episode of UTI in the postoperative period.

## COMMENT

Urethral injury associated with pelvic fracture is seen periodically at trauma centers. Male urethral injury is present in 10% of pelvic fractures[1], while in females, only 4–6% have an associated urethral injury due to the anatomically shorter and more mobile urethra that travels behind the bony arch of the pubis[2,3]. Urethral injury in the absence of pelvic fracture is exceedingly rare. McCool and Joseph previously reported a complete urethral and bladder neck disruption without pelvic fracture in a 4-year-old unrestrained female in a motor vehicle accident[4]. The authors attributed this unique case to the childhood location of the distended bladder outside of the deep pelvis. To our knowledge, this is the first reported case of an adult female urethral injury without an associated pelvic fracture. The mechanism for urethral injury in our patient may have been a full bladder at the time of impact that, when combined with the torque from the seatbelt restraint, caused a traumatic, full-thickness, urethral vertical tear with involvement of the bladder neck.

Urethral injury can be longitudinal (incomplete) lacerations or avulsions (complete), and most are accompanied by anterior vaginal laceration[2,3,4]. Most authors favor primary repair over delayed repair whenever possible, while some distal urethral injuries may be left unrepaired with a subsequent hypospadiac urethral meatus[2,3,4,5,6]. Depending on the extent of urethral injury, repair may be performed vaginally, transabdominally, or combined[2]. Our patient’s longitudinal urethral injury was closed primarily in three layers. Proper exposure is important and was accomplished in our patient by dorsal lithotomy positioning after fixation of orthopedic injuries. Women should be followed postoperatively to address lower urinary tract symptoms and sexual dysfunction after repair.
